# Health Complaints Associated with Poor Rental Housing Conditions in Arkansas: The Only State without a Landlord’s Implied Warranty of Habitability

**DOI:** 10.3389/fpubh.2016.00263

**Published:** 2016-11-23

**Authors:** Ashley E. Bachelder, M. Kate Stewart, Holly C. Felix, Neil Sealy

**Affiliations:** ^1^University of Arkansas for Medical Sciences Fay W. Boozman College of Public Health, Little Rock, AR, USA; ^2^Arkansas Community Institute, Little Rock, AR, USA

**Keywords:** landlord, tenant, landlord–tenant law, habitability, housing

## Abstract

Arkansas is the only U.S. state that does not have a landlord’s implied warranty of habitability, meaning tenants have a requirement for maintaining their rental properties at certain habitability standards, but landlords are not legally required to contribute to those minimum health and safety standards. This project assessed the possibility that this lack of landlord responsibility affects tenants’ perceived health. Using surveys and interviews, we collected self-reported data on the prevalence and description of problems faced by renters who needed household repairs from their landlords. Of almost 1,000 renters, one-third of them had experienced a problem with their landlord making needed repairs; and one-quarter of those had a health issue they attributed to their housing conditions. Common issues included problems with plumbing, heating, or cooling systems, and pest or rodent control. Reported health problems included elevated stress levels, breathing problems, headaches, high blood pressure, and bites or infections. Hispanic respondents and those with less than a high school education were both significantly more likely to report problems with their landlords not making repairs as requested. These data suggest that the lack of landlord requirements may negatively impact the condition of rental properties and, therefore, may negatively impact the health of Arkansas renters.

## Introduction

We spend a great deal of time in the spaces in which we live, and it has been long understood that housing conditions have a significant impact on health status (1–3). Individuals who live in substandard housing are more likely to encounter material or physical hazards such as pest infestations, mold, leaks and dampness, poor ventilation, noise pollution, injury hazards, extreme temperatures, exposure to lead, or other poisoning and carcinogenic air pollutants or allergens that may trigger negative health effects (4–7). Such exposures may increase many health risks through multiple pathways and through both acute and chronic responses including headaches, fever, nausea, vomiting, changes in blood pressure, myocardial infarction, injuries, mental and psychological distress, asthma, respiratory infections, obesity, diabetes, decreased neurological functioning, some types of cancer, and overall mortality (1–7). The same populations that already carry a greater burden of disease and illness are likely to disproportionally be impacted by home health hazards. Racial and ethnic minorities, the poor, LGTBQ individuals, individuals with disabilities, immigrants, and other marginalized groups are more likely to live in unsafe or unstable housing, lack necessary financial resources to change their living situations, and/or are more likely to face discrimination when searching for adequate housing (1, 3, 7–12).

An estimated 32% of the U.S. population, or nearly 100 million residents, currently rent the homes in which they reside (13); meaning landlords have a shared responsibility, with the tenant, to ensure safe and healthy living quarters. Model legislation introduced in 1972 called the Uniform Residential Landlord Tenant Act (URLTA) established a balanced framework for landlord–tenant laws which outlined rights and responsibilities of both parties for maintaining basic minimum habitability standards. For landlords, those responsibilities include things such as adhering to applicable building codes, maintenance of electrical, plumbing, and heating systems, and providing receptacles for garbage storage and removal. Tenant responsibilities include the reasonable use of property, proper disposal of waste, no deliberate or negligent intent to destroy the property, etc. (14). Furthermore, the Act established an “implied warranty of habitability” – an implied standard and requirement that landlords maintain habitable living conditions and perform basic property repairs even if the lease contract does not expressly dictate exact details (15). The vast majority of states have adopted some form this model legislation (15).

Currently, there is no research that links if and how habitability laws impact housing conditions, and by extension, tenant health status. It is difficult to evaluate the direct impact of the URLTA or the implied warranty of habitability, in part due to the fact that states have not uniformly adopted the URLTA in a singular format. In a review of the strength and variability of habitability laws nationwide, Willis found great variability in the years the laws were adopted (ranging 1972–2008), the specific URLTA habitability elements approved (e.g., states may have adopted elements related to waste removal, but not water and heat), the distribution of landlord and/or tenant responsibilities adopted (e.g., seven states adopted landlord requirements only), and the regulatory authority and strategies for enforcement. In his analysis, he found Southern states to have, overall, lower responsibility delegated to the landlord for maintaining habitable environments and greater requirements and burden placed on the tenant for doing so (16). Moreover, the lack of an integrated database for housing quality indicators and health status indicators also presents a challenge in measuring the direct impacts that housing improvements may cause on population health (3). However, given the growing base of evidence regarding housing improvements and their positive associations with health improvements and decreased health risks (17–20), it can be inferred that new housing and public policies, such as strengthened and enforced habitability laws, will provide a pathway for improved tenant health *via* improved physical home conditions (21, 22).

Arkansas is the only state in the nation that has not adopted any of the URLTA landlord requirements. When the state first considered adopting a version of the URLTA in 2007, the legislature accepted the tenant requirements only and rejected all landlord elements, leaving tenants with little to no recourse against landlords who do not expressly include the landlord’s maintenance responsibilities in a lease agreement (23).

The legislature has considered various habitability bills during each session since 2007. The most recent attempt in 2015 would have established specific property maintenance requirements and would have prohibited landlords from retaliating against tenants who request repairs. Tenants would also have been given a legal mechanism to terminate a lease without financial or legal penalty if the landlord does not maintain the property to the minimum standards (24).

Despite pro-adoption recommendations from a commission specifically created to study the topic and support from many allied groups, the 2015 bill failed to pass out of legislative committee. Arguments against the bill included concern over increasing rent prices, hindering housing development, increasing costs and liabilities for landlords, and a general dislike for increased business regulations. Child advocacy groups have since stated that this bill was a missed opportunity for advancing equity for racial minority and low-income communities.

Thirty-four percent of Arkansas housing units, or approximately 450,000, are rental ones (25), which signals the sizable population that more comprehensive habitability laws may impact. The possible health impacts that substandard housing has on individuals, and how a change in policy may mitigate or worsen those impacts, has been absent from Arkansas’s warranty of habitability debates. As the field of health impact assessment (HIA) grows in the U.S., this type of analysis that considers how economic, social, or environmental factors outside traditional health or health-care sectors has the potential to bring new information to the decision and policy-making process (26, 27). To date, only about a dozen known, formal HIAs in the U.S. have been carried out which focus directly on housing policies such as building health and safety codes or structural design (28, 29).

This paper shares formative data collected through surveys and interviews of Arkansas renters focused on how Arkansas’s current landlord–tenant laws, specifically the lack of an implied warranty of habitability indicating landlord responsibilities, may contribute to unsafe and unhealthy housing conditions and cause or exacerbate poor health. These data have since been used by community and advocacy organizations in obtaining funding for an HIA on the state’s habitability law to be completed before the next legislative session (30).

## Materials and Methods

This project was completed, in part, as an unfunded, student service learning project in partnership between a racial and ethnic health disparities graduate course at the University of Arkansas for Medical Sciences in Little Rock, Arkansas and Arkansas Community Organizations (ACO), a community-based non-profit organization also located in Little Rock.

### The Instruments

#### Surveys

Survey questions, developed by course instructors and the community partner, included two screening questions asking if the participant currently or had ever rented in Arkansas, awareness of Arkansas’s landlord–tenant laws, frequency of problems with the condition of rental property, an inventory of those maintenance issues, handling of issue by landlord, and attributable health effects. English and Spanish surveys were available.

#### Interview Guide

A semi-structured interview guide, also created by instructors and the community partner, was created with three domains: household conditions, relationship with landlord, and health impacts.

### Recruitment and Data Collection

#### Surveys

The majority of surveys were collected in-person by students in the service learning course. They served as volunteer greeters at an ACO-operated free tax filing site in Little Rock (the state capital and largest city). During the client intake process, the student explained the purpose of the survey and explained it was voluntary and would not affect their ability to receive tax or other services. If they agreed to participate, they were given the survey to self-administer unless they required assistance due to illiteracy or other accessibility issues. Surveys were also collected at the second ACO-operated free tax filing site in Pine Bluff, a smaller and predominately low-income city in southeast Arkansas, as part of the intake paperwork. At this location, however, there were no students to introduce the purpose, answer questions, or administer if needed. The survey was rather part of the paperwork to complete, including written instructions. Some students also collected surveys *via* convenience sampling at other locations and events (i.e., college fairs and health fairs). They approached individuals and explained the purpose of the survey, and participants completed the survey independently (unless needing assistance). Data collection took place in February and March 2015.

#### Interviews

Survey participants voluntarily provided their contact information if they were interested in participating in an interview. Participants who reported problems with both their housing conditions and their landlord and who provided their contact information were recruited by phone by a course instructor and in-person interviews with that instructor were arranged. Participants signed a consent form and were given $25 cash. Interviews were completed in English, audio-recorded, and notes were typed on a laptop.

### Data Analysis

#### Surveys

Paper surveys were scanned using ABBYY FlexiCapture technology. One instructor conducted quality control checks of every survey by comparing the entered data to the original version and manually corrected data that did not scan accurately. These data were then transferred into STATA for analysis. Analysis included frequencies and descriptive statistics, bivariate analysis of outcomes by race/ethnicity, and logistic regression to identify demographic characteristics associated with having problems with a landlord making repairs. Respondents’ racial/ethnic identity was defined as follows: those who identified as Hispanic; non-Hispanic respondents who identified as Black; and non-Hispanic respondents who identified as White. Gender, race/ethnicity, income, and education variables were used in logistic regression models. Initial analysis examined the overall percent of respondents who needed a repair and had a problem with the landlord making the repair. Subsequent analysis focused on determining if race/ethnicity was associated with having a problem with the landlord.

#### Interviews

Written transcripts were prepared and selected quotes are presented to illustrate key points.

This project was reviewed by the UAMS Institutional Review Board and determined not to be human subjects’ research.

## Results

### Participants

#### Surveys

Just over 1,100 (*n* = 1,108) surveys were collected. The 157 respondents who reported never having rented in Arkansas were removed from analysis, leaving 951 completed surveys from current or past renters. Table 1 summarizes their demographics. The mean age was 42 years and the range spanned from 16- to 81-year olds. The majority were females (62%) and Black or African-American (71%). Nearly one-fifth (18%) were identified as Hispanic. Two-thirds of the sample (66%) had a combined household income under $30,000. Just over 81% had a high school education or higher, and 11% held a Bachelor’s degree or higher.

**Table 1:** **Survey participant demographics**.

*N* = 951	*N*	%
**Age (*n* = 871)**		
Mean age	42.19	–
16–24	85	9.76
25–34	206	23.65
35–44	210	24.11
45–54	180	20.67
55–64	143	16.42
65–74	41	4.71
75 or older	6	0.69
**Gender (*n* = 856)**		
Female	533	62.27
Male	323	37.73
**Race/ethnicity (*n* = 894)**		
White, non-Hispanic	102	11.41
Black or African-American, non-Hispanic	634	70.92
Hispanic	158	17.67
**Combined family income (*n* = 830)**		
Less than $10,000	184	22.17
$10,000–$19,999	178	21.45
$20,000–$29,999	186	22.41
$30,000–$39,999	118	14.22
$40,000–$49,999	111	13.37
$50,000–$74,999	42	5.06
$75,000 or more	11	1.33
**Education (*n* = 871)**		
Some high school	168	19.29
High school or GED	261	29.97
Some college	252	28.93
Associate degree	94	10.79
Bachelor’s degree	63	7.23
Graduate/professional degree	33	3.79

#### Interviews

Twenty individuals who provided their contact information and met the interview criteria were contacted to request an interview, and five of them accepted and were interviewed. Interviewees ranged from ages 22- to 60-year olds, included three females and two males, three African-Americans, one White, and one Hispanic person, income levels between $10,000 and $40,000 and education including Associate degrees, some college, and high school levels.

**Figure 1:**
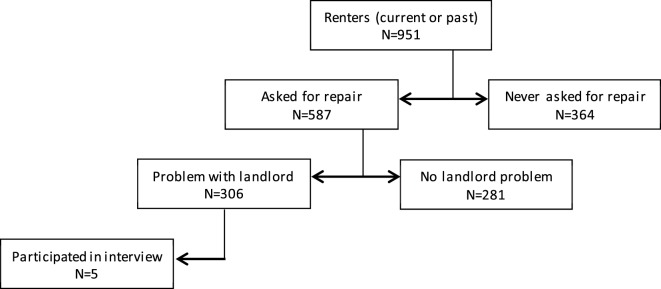
**Survey response frequencies**.

About one-third of the sample (32%) reported needing a repair in their home and having a problem with the landlord making those repairs. Figure 1 illustrates the frequencies for all requested repairs and landlord troubles. Sixty-eight percent of all respondents were not aware that the state did not have expressed requirements for landlords to maintain basic standards.

### Risk Factors for Problems with Landlord

We compared those individuals who asked for a repair and were having problems with the landlord with those who needed a repair but did not have problems with the landlord. Being Hispanic and having less than a high school education were both significantly associated with having a problem with the landlord. Hispanic respondents were 41% more likely to report problems compared to White respondents (RR = 1.413; *p* = 0.024). Compared to people with more than a high school education, those with less than high school education were or 33% more likely to indicate problems with the landlord (RR = 1.328; *p* = 0.017). There were no significant differences by gender or household income. See Table 2 for detailed regression results.

**Table 2:** **Regression analyses for having a problem with the landlord**.

Variable	Risk ratio	*p*-Value
Black	1.056	0.655
Hispanic	1.413	0.024
Male	1.088	0.358
Less than high school education	1.329	0.017
High school education	0.976	0.800
Household income less than $10,000	1.092	0.447
Household income between $10,000 and $29,999	1.027	0.701

*Comparisons of variables made with multivariate logistic regression and calculation of relative risk ratios*.

### Description of Problems and Responses

Nearly three-quarters of participants (73%) had experienced problems with their landlord one or two times; although more than one-fifth (21%) reported problems three or four times. Almost half (45%) of participants who reported a problem paid between $400 and $599 in monthly rent, and about a fourth paid less than $400 (25%) or between $600 and $799 (23%). The majority (77%) who reported problems resided in a private rental without subsidized funding.

The remaining data focus on the most recent problem that participants had. The most common maintenance issue reported was plumbing related (51%), followed by heating or cooling (42%), pests or rodents (36%), windows or doors (25%), and electric (24%).

When asked what they did about the problem, the most common response was that they asked the landlord to fix the problem more than once (73%), followed by moving (38%), asked to fix only once (19%), and tried to fix problem themselves (19%). Fifty percent of Hispanics reported moving, a significantly higher rate than 30% of Whites and 33% of Blacks who moved (*p* = 0.019).

For landlord responses to requested repairs, the landlord eventually fixed the problem for 55% of respondents. Twenty percent of respondents reported being threatened with eviction, 10% reported that the landlord ignored the request, and 9% experienced verbal abuse. Almost half (45%) of Hispanic respondents were threatened with eviction versus only 10% of Whites and 10% of Black respondents (*p* = 0.00). Similarly, 17% of Hispanic respondents experienced verbal abuse from the landlord versus 7% of Whites and 6% of Blacks (*p* = 0.029).

### Health Impacts

Of those who had problems with their landlord making repairs, one-quarter (25%) perceived that the problems impacted their health or the health of their family. Increased stress was the most frequently cited response (69%), followed by 46% who reported breathing problems, 37% indicated headaches, 27% with blood pressure problems, 22% had insect or animal bites, and 16% reported skin problems. Of those reporting health problems, 27% reported having to seek care from the emergency room or doctor. In the qualitative interviews, participants shared stories about ant infestations (despite constant cleaning) which triggered emergency department visits for a young child; severe depression and suicidal ideation for a single mother worried about her children; and increased stress and spikes in hypertension, among others. Table 3 includes illustrative quotes from participant interviews describing these and other issues.

**Table 3:** **Interview quotes illustrating description of problems, tenant reported landlord response, and health impacts**.

Description of problems	“For the whole time we were there we never had a key to the front door. The type of lock would require a special configuration and they [the landlord] weren’t going to pay for it. We had to use the side door and could only use the front door if someone was home. And the side door was faulty” “The termites were coming through the sockets, vents, cracks in the wall. We were sucking them up with a vacuum. We would wake up and they’d be in our face. Thousands of them. It’s hard to sleep when you’re breathing in termites and you wake up and your bed is full of them. We went out and got our own spray, but you have to spray and then sleep there” “The roaches were just unbelievable. They were all in the bed – I had to pick them out of my baby’s hair. They were in our food. We were coughing a lot” “It was the dead of winter and freezing when the hot water heater went out. I had a 1-year-old then. I asked for her to fix it immediately but she didn’t. I paid my neighbors to use their shower”

Tenant reported landlord responses	“You had to request repairs online. Usually all we got was a phone call and a ‘promise’ but they never did anything. Communications were just lip service. Any repairs you make you can’t deduct from the rent, even with receipts” “The only repairs she’s even given me are superficial ones, like repainting the walls to make the décor look clean” “The owner had over 25 properties – he just didn’t have the finances or the desire to put anything into the houses he has. He and his wife live in a Winnebago – they travel a lot. They use the property management company to deflect the heat and not really do anything”

Health impact	“In regards to the ants, being at work was just enough time for them to mingle around to my kid’s bedroom. After getting off of a long day, I had to move the bed, vacuum, and change sheets before I could put her to sleep. It was something I had to do to protect my child from ant bites. I had to take my 5-year-old daughter to the ER one time. She developed welts and suffered an allergic reaction to the ant bites. They itched so bad, they later got infected” “It causes stress and blood pressure. You’re reluctant to have company because of the embarrassing conditions. Around that time I started going to the doctor more often. That was when I started feeling that I had to find out what was up with me, I was not quite myself – my energy level was down” “Someone called DHS so they came to look at the place. They were threatening to take my daughter if this thing didn’t get fixed. My landlady didn’t do anything. It was so bad I had depression. After [DHS] came and talked to me about the house I wanted to kill myself. I got help for that. I had to go to the ER and they kept me there for about 3 days. My depression is still not as controlled as it needs to be”

## Discussion

This project suggests that experiencing problems with getting their landlords to make needed repairs may be common among low-income Arkansas renters, particularly for Hispanic renters and those with low education levels. Those who reported landlord problems often experienced health issues that they attributed to the condition of their rental property. For Hispanic respondents, health issues were not the only problem, as they were significantly more likely to experience verbal abuse, be threatened with eviction, and move than White or Black renters.

Some sampling limitations exist. The surveys were collected through a non-random convenience sample with geographic areas not representative from the entire state. The sample may not be representative of all renter experiences, given its high proportion of respondents of low socioeconomic status, women, and racial and ethnic minorities. However, census data show lower income households to be more concentrated in renter-occupied housing units. For example, in our sample, 44% of respondents had an annual combined household income under $20,000. U.S. Census data show 37% of Arkansas renters to fall within the same income categories (25). Similarly, in our sample, 49% of respondents reported an educational attainment of high school diploma or less, and Census data show 48% of Arkansas renters to have an equivalent education status (25). We intentionally oversampled at locations that serve low-income individuals, which accounts for the high percentage of African-American respondents in our sample given the known fact that African-Americans are disproportionally represented in low-income categories. Given these data, we believe our sample is fairly representative of Arkansas renters.

This project did not assess the health of participants living in high quality or decent housing conditions or where repairs were made in a timely manner. These data would have allowed us to determine whether health problems are more common and/or worse in those who do not get repairs compared to those who did not need repairs and/or those whose landlords make repairs when needed. Had these data been collected, it may have allowed us to more definitively distinguish the role of the landlord in the health issues experienced. For this reason, we view our findings as suggestive, and this formative research may be used to guide further studies on this topic.

We are not aware of other projects that have studied how Arkansas’s landlord–tenant laws impact the health of tenants. The data presented in this paper illustrate some suggested health impacts from substandard housing conditions which may be linked to the landlord–tenant law, but they do not explore all possible health impacts or any unintended consequences that adopting a comprehensive implied warranty of habitability may cause. Additional pathways, such as considering how reform of the landlord–tenant law may impact housing stability, could present other indirect health impacts that may be negative or positive. Enforcement mechanisms for habitability policies also need attention to ensure that any changes to the laws and practices would be adhered to and a true analysis could show its impact on health and other indicators of well-being. Using these data and other experiences, ACO and its research partner, the Arkansas Community Institute, successfully secured funding to plan and conduct a comprehensive HIA to further study these and other factors regarding how the current and possible modifications of Arkansas landlord–tenant laws impact vulnerable communities.

Information collected through the HIA process will inform the advocacy efforts of community-based organizations aimed at the Arkansas legislature for when another habitability bill is proposed. The objective of the HIA process is to facilitate direct tenant and landlord participation in the policy-making process, with a goal of ensuring future legislation addresses issues that are most relevant to felt community needs.

## Ethics Statement

This project was reviewed by the University of Arkansas for Medical Sciences Institutional Review Board and determined not to be human subjects’ research and therefore not subject for full review.

## Author Contributions

AB was the project coordinator for this student project. She conceived the project idea with MS and NS. She designed the project, oversaw student data collection, ran statistical analysis, managed data, prepared article drafts, and made article revisions. MS conceived the project idea with AB and NS. She assisted in data analysis, reviewed drafts, and provided feedback for revisions. HF led data analysis process, reviewed article drafts, and provided feedback for revisions. NS conceived the project idea with AB and MS. He assisted with data analysis, data interpretation, reviewed article drafts, and provided feedback for revisions.

## Conflict of Interest Statement

The authors declare that the research was conducted in the absence of any commercial or financial relationships that could be construed as a potential conflict of interest.
